# Zika Virus Infection and Microcephaly: A Case-Control Study in Brazil

**DOI:** 10.5334/aogh.2394

**Published:** 2019-08-28

**Authors:** Sabrina Gabriele Maia Oliveira Rocha, Luciano Lima Correia, Antônio José Lêdo Alves da Cunha, Hermano Alexandre Lima Rocha, Álvaro Jorge Madeiro Leite, Jocileide Sales Campos, Tereza de Jesus Pinheiro Gomes Bandeira, Lucas Silveira do Nascimento, Anamaria Cavalcante e Silva

**Affiliations:** 1 Federal University of Ceará, Community Health Department, Fortaleza, Ceará, BR; 2 Federal University of Rio de Janeiro, Rio de Janeiro, BR; 3 Christus University Center (Unichristus), Fortaleza, Ceará, BR

## Abstract

**Background:**

Brazil presented an alarming number of newborns with microcephaly in the years 2015 and 2016. The investigation of the cases raised the suspicion of the association of these cases with maternal infections by the zika virus. Also, in 2015, there was an epidemic of zika virus infection in Brazil, reinforcing this hypothesis.

**Objective:**

The objective of this study was to identify factors associated with the diagnosis of microcephaly in newborns, including zika virus infection.

**Methods:**

We conducted a case-control study. The cases were defined as children who received clinical and imaging diagnosis of microcephaly, born after October 2015 in Ceará, Brazil, which recorded the highest number of microcephaly cases in Brazil during the outbreak. The cases were identified in medical records of public and private maternity hospitals and in child development stimulation clinics tracked until June 2017. Epidemiological, clinical, and socioeconomic variables were collected, visiting their homes and confirming data from their medical records. Controls were children without microcephaly identified in the vicinity of the residence of each case. Logistic regression models were used to control confounding.

**Findings:**

We evaluated 58 cases and 116 controls. The odds of having a baby with microcephaly was 14 times higher among mothers who had zika virus infection (p < 0.001), after multivariate analysis. Arboviruses infections symptoms, as fever (p = 0.220), skin change (p < 0.001), and joint pain (p = 0.002) also demonstrated an association with microcephaly.

**Conclusions:**

Maternal infection zika virus was associated with a diagnosis of microcephaly. Our study contributes to the investigation of the epidemiological factors associated with the diagnosis of microcephaly.

## Introduction

Since October 2015, Brazil has had an alarming number of suspected cases of newborns with microcephaly. During that period, the concomitance with the zika virus infection epidemic in the country aroused the suspicion of an association between these conditions. Therefore, the World Health Organization (WHO) declared a public health emergency in February 2016 [1,2].

Microcephaly is defined as a clinical sign of congenital malformation in children presenting occipitofrontal circumference two standard deviations lower than the mean compared to children of the same age, sex, and ethnicity [3]. It is a rare condition, with an incidence of 5.9 cases per 10,000 live births in the United States of America [4], and its diagnosis can be confirmed as delayed brain development identified by imaging tests, such as intrauterine ultrasonography (US) or computer tomography (CT) after childbirth.

This congenital malformation has many causes, such as genetic factors and congenital infections, and it is also associated with social factors. Among the congenital infections, cytomegalovirus [5], rubella [6,7,8], toxoplasmosis [9], and syphilis [10] stand out as the leading agents. Low education, alcohol abuse, and inadequate prenatal care are also associated with microcephaly [4]. However, a study revealed that 41% of microcephaly cases do not have a definitive cause and are categorized as idiopathic cases [11].

Current studies have confirmed the biological plausibility of a link between zika virus and microcephaly: case reports have confirmed stillbirths infected by zika virus [12]; other studies have shown brain tissue tropism caused by zika virus [13], the vertical transmission of zika through the placenta [12,14], an ecological association [15], and a higher incidence of malformations in pregnancies with confirmed Zika infections [16].

According to many authors [17,18], studies must be developed to obtain more information about this malformation outbreak, including the temporal patterns of the complications after the epidemic waves [19] and its repercussion in the present and future generations.

This present case-control study was performed in the state of Ceará, Northeastern Brazil, to contribute to the understanding of the association between zika virus infection and microcephaly and to draft new political-administrative measures for its management.

## Methods

### Study design

This exploratory case-control study intended to identify protective factors and risk factors for microcephaly in the state of Ceará, Northeastern Brazil – the Brazilian region with the highest incidence of microcephaly.

The cases were defined as children with a clinical diagnosis of microcephaly with an imaging diagnosis of microcephaly performed by computed tomography (CT) or intrauterine ultrasonography (US). The children were born after October 2015, correlating the first trimester of pregnancy with the period of the zika virus epidemic. These newborns were identified not only in public and private maternity records but also in reference services for tracking and infant development until June 2017.

The controls were children without microcephaly, showing a cephalic perimeter larger than the 95% percentile for age according to WHO curves, as well as no signs or symptoms of congenital malformations. They were identified near the residence of each child in the group of cases, with the same age or within a two-month range, ensuring the homogeneity of temporal and environmental exposure in both groups.

### Study setting and population

The study population included children of both sexes born from October 2015 until June 2017 who live in the State of Ceará.

Ceará is one of the poorest States in Brazil, ranking 17th in the Human Development Index (0.682) out of the 27 States in the country [20]. Ceará is in the northeastern region of Brazil, and approximately 93% of its land area of 148,000 km² presents a semiarid climate [21]. With a population of 8 million inhabitants [18], and an annual average of 128,000 live births [21], the state is the fourth in total confirmed microcephaly cases (137 cases) and the first in total of fetal and neonatal deaths resulting from microcephaly (24 cases, 16.9%) [22]. The territory of Ceará comprises 184 municipalities, and there have been confirmed cases of microcephaly in 53 (28.8%) of them [22].

### Study sample

This study of prevalent cases aimed to enroll a sample of children with a confirmed diagnosis of microcephaly born in Ceará through June 2017. The control group of children was recruited using a ratio of two controls for each case to increase the sample power [23]. The data were collected from 58 randomly selected cases, and from this sample size, it is possible to detect an odds ratio (OR) of 2.3, with a 5% significance level and 80% sample power for exposure with 28% prevalence in the control group.

### Data collection

Children diagnosed with microcephaly were identified by the infant care network, collecting mothers’ names and their respective addresses. We obtained the data from these units: the Assis Chateaubriand Maternity School – MEAC, the main public maternity of Ceará; the Albert Sabin Children’s Hospital – HIAS, reference for infant diseases of greater complexity in the Northeastern region of Brazil; and the Core of Treatment and Precocious Stimulation – NUTEP, reference service for children’s precocious stimulation in Ceará.

The data collection was conducted in the children’s households through a questionnaire containing data from the mothers, the children, and their families. The field research team composition was one supervisor and six interviewers, all of them with nursing or physiotherapy degrees and with proper training for the interview and anthropometric measurements. The team made five attempts to find the mother and her child at home to collect the data.

The anthropometric measurements of the children (cases and controls) were carried out just after the mother’s interview. The occipitofrontal perimeter was measured using a retractable inelastic measuring tape with a security lock (1.5 m in size and 1 mm precision). A portable digital scale measured the weight with a capacity of 180 kg and an accuracy of 100 g, and the weight was obtained with the child on the mother’s lap followed by subtracting the mother’s weight. The measurement of the children’s length was performed with the child lying down on an infant anthropometer, with a size of 1.5 m and precision of 1 mm.

### Quality control

To reduce memory bias, we checked the data from the pregnancy and the prenatal care in the Pregnancy Booklet (official medical record of pregnant women in Brazil), which is provided by the maternity ward presenting the gestation registers and the prenatal consultations. Children’s data, such as cephalic perimeter and weight at birth, were confirmed by the Child’s Health Booklet (official medical record for children in Brazil), which is provided by the Ministry of Health to all children at birth.

The scales were calibrated according to the producer’s indication. A random sample of 10% of the questionnaires was repeated by the field supervisor to identify possible errors and fill the gaps to validate the quality of the data collection.

The data was double entered in the software program to solve typing errors or data loss.

### Variables

The dependent variable, the outcome, refers to the presence or absence of microcephaly, with a confirmed diagnosis by clinical or imaging criteria (CT scan or intrauterine US) in the hospital or maternity ward, following the government protocol [24].

The independent variables were grouped and categorized into the blocks described below.

Diseases during pregnancy included the following – self-reported and confirmed by the pregnancy booklet: zika virus infection diagnosed by a medical doctor, joint pain, clinical syndromes with a skin rash, eye pain, cytomegalovirus, syphilis, rubella, toxoplasmosis, AIDS, flu or other viruses.

The exposures during pregnancy included the following – self-reported: if one had contact with solvents, inks, varnishes, poisons, hair dye or smoothing agents with ammonia; if the mother underwent tests with radiation during pregnancy (X-ray or CT), magnetic resonance imaging, tobacco use, and alcohol consumption.

### Data analysis

Bivariate analysis was performed, calculating the proportions of categorical variables and measures of central tendency and dispersion for numeric variables. The differences found between the cases and controls were evaluated for statistical significance using the chi-square test for categorical variables, students’ t-test was used for numeric variables with normal distribution, and the Mann-Whitney test was used for numeric variables with nonnormal distribution. The Kolmogorov-Smirnoff test evaluated the normality of variables. Additionally, if one cell registered fewer than five cases, we used Fisher’s exact test.

For the control of confounders, the variables with a p-value lower than 0.1 were included in a logistic regression model with adjusted odds ratio calculations.

Analyses were performed using SPSS software, version 17, SPSS Inc., considering a p-value ≤0.05.

### Ethical aspects

The study was submitted to the Ethics Committee through the Brazil Platform, following all the norms of the 466/2012 Resolution of the National Council of Health of the Ministry of Health [25], and it was approved under protocol number 1.449.427. All the mothers who agreed to participate in the research signed informed consent forms.

## Results

### Children’s baseline characteristics

The median age and sex of cases and controls were not different (nine months and 57% male). However, there was a difference between cephalic perimeters (35 and 31 centimeters), birth weights (2.67 and 3.22 kg), and gestational ages, as shown in Table 1.

**Table 1:** Baseline and birth characteristics of the sample.

		Condition						p value
		
		Case		Control		Total		

		n or median	% or interquartile	n or median	% or interquartile	n or median	% or interquartile	

Sex	Male	29	50.9%	69	60.0%	98	57.0%	0.194
	Female	28	49.1%	46	40.0%	74	43.0%	
Age in months		6.50	4.0–21.0	11.00	4.0–20.0	9	4.0–20.0	0.474
Birth weight		2.67	2.3–3.0	3.22	2.8–3.7	3.010	2.6–3.4	<0.001
Cephalic perimeter		31.00	29.5–32.0	35.00	34.0–35.5	33.0	31.0–35.0	<0.001
Timing of birth	Normal	45	77.6%	94	81.7%	139	80.3%	0.025
	Preterm	12	20.7%	9	7.8%	21	12.1%	
	Post term	1	1.7%	9	7.8%	10	5.8%	
	Not specified	0	0.0%	3	2.6%	3	1.7%	

### Diseases during pregnancy

The mothers of the patients had two-times more fevers than the mothers of the controls (p-value = 0.022), and these cases of illness were earlier in the first trimester in cases (p = 0.032). Among the women who received a diagnosis during pregnancy, there was a predominance of zika virus syndrome in the pregnant women from the cases, while the majority of the mothers of the controls had urinary tract infections or conditions other than zika (p = 0.040). The signs and symptoms were muscular pain, joint pain, weakness, and skin changes, also showing a difference between the groups (0.028, 0.002, 0.041, <0.001, respectively). Mothers of the cases showed almost twice as many reports of insect bites during pregnancy. The odds ratio of having zika virus of the cases compared to the controls was 10.35 (p-value < 0.001) (Table 2).

**Table 2:** Ratios of diseases presented by the mother during pregnancy in cases and controls.

		Condition				p value

		Case		Control		

		n or median	% or interquartile	n or median	% or interquartile	

Fever	Yes, once	16	28.1%	16	13.9%	**0.022**
	Yes, more than once	9	15.8%	11	9.6%	
	No fever	32	56.1%	88	76.5%	
Gestation month of fever		3	2.0–6.0	5	4.0–7.0	**0.032**
Days of fever		2	1.0–3.0	3	3.0–5.0	0.057
Fever intensity	Low	11	50.0%	6	26.1%	0.151
	Moderate	8	36.4%	9	39.1%	
	High	3	13.6%	8	34.8%	
Any appointment with a physician	Yes	18	58.1%	26	66.7%	0.449
	No	12	38.7%	13	33.3%	
	Do not remember	1	3.2%	0	0.0%	
Diseases during pregnancy	Other	8	47.0%	12	46.1%	**0.040**
	Urinary infection	1	5.2%	12	46.1%	
	Zika	8	47.0%	2	7.6%	
Headache intensity	Low	8	15.1%	16	17.4%	0.865
	Moderate	10	18.9%	20	21.7%	
	High	14	26.4%	19	20.7%	
	None	21	39.6%	37	40,2%	
Stiff neck	Yes	5	9.6%	14	14.4%	0.599
	No	47	90.4%	81	83.5%	
	Do not remember	0	0.0%	1	1.0%	
Eye pain	Yes	12	22.2%	14	15.2%	0.227
	No	41	75.9%	78	84.8%	
	Do not remember	1	1.9%	0	0.0%	
Photophobia	Yes	10	18.5%	13	13.5%	0.417
	No	44	81.5%	83	86.5%	
Seizures	Generalized	2	3.7%	1	1.0%	0.527
	Focal	2	3.7%	4	4.1%	
	No	50	92.6%	92	94.8%	
Disorientations	Yes	0	0.0%	5	5.2%	0.172
	No	54	100.0%	90	93.8%	
	Do not remember	0	0.0%	1	1.0%	
Amnesia	Yes	2	3.8%	5	5.4%	0.678
	No	51	96.2%	87	93.5%	
	Do not remember	0	0.0%	1	1.1%	
Behavioral changes	Yes	13	24.1%	29	30.2%	0.422
	No	41	75.9%	67	69.8%	
Dyspnea	Yes	16	30.2%	27	28.7%	0.851
	No	37	69.8%	67	71.3%	
Precordialgia	Yes	4	7.4%	14	14.4%	0.202
	No	50	92.6%	83	85.6%	
Dysarthria	Yes	0	0.0%	6	6.3%	0.062
	No	53	100.0%	89	93.7%	
Epigastralgia	Yes	10	18.5%	17	17.5%	0.879
	No	44	81.5%	80	82.5%	
Diarrhea	Yes	5	9.4%	10	10.3%	0.864
	No	48	90.6%	87	89.7%	
Nausea	Yes	33	61.1%	58	60.4%	0.933
	No	21	38.9%	38	39.6%	
Sore throat	Yes	6	11.1%	21	21.6%	0.105
	No	48	88.9%	76	78.4%	
Cough	Yes	13	24.1%	23	24.0%	0.987
	No	41	75.9%	73	76.0%	
Back pain	Yes	27	50.0%	58	59.8%	0.245
	No	27	50.0%	39	40.2%	
Muscle pain	Yes	14	26.9%	12	12.5%	**0.028**
	No	38	73.1%	84	87.5%	
Joint pain	Yes	23	42.6%	19	19.6%	**0.002**
	No	31	57.4%	78	80.4%	
Difficulty moving	Yes	12	22.6%	14	14.9%	0.237
	No	41	77.4%	80	85.1%	
Joint edema	Yes	17	31.5%	25	26.0%	0.600
	No	37	68.5%	70	72.9%	
	Do not remember	0	0.0%	1	1.0%	
Paralysis	Ascending	1	1.9%	3	3.2%	0.629
	No	53	98.1%	91	96.8%	
Weakness	Generalized	13	24.1%	9	9.6%	**0.041**
	Focal	4	7.4%	13	13.8%	
	No	37	68.5%	72	76.6%	
Conjunctivitis	Yes	0	0.0%	2	2.1%	0.290
	No	52	100.0%	92	97.9%	
Bleeding	Yes	10	19.6%	16	17.2%	0.720
	No	41	80.4%	77	82.8%	
Skin changes	Yes	25	48.1%	14	14.7%	**<0.001**
	No	27	51.9%	81	85.3%	
Thick skin	Yes	11	42.3%	5	27.8%	0.325
	No	15	57.7%	13	72.2%	
Spot with a border	Yes	3	12.0%	0	0.0%	0.128
	No	22	88.0%	18	100.0%	
Erythema	Yes	17	68.0%	7	38.9%	0.058
	No	8	32.0%	11	61.1%	
Vesicles	Yes	1	4.0%	0	0.0%	0.391
	No	24	96.0%	18	100.0%	
Itchy spots	Yes	11	44.0%	6	33.3%	0.480
	No	14	56.0%	12	66.7%	
Petechiae	Yes	16	64.0%	7	38.9%	0.103
	No	9	36.0%	11	61.1%	
Bruises	Yes	3	12.0%	0	0.0%	0.128
	No	22	88.0%	18	100.0%	
Stings during pregnancy	Yes	17	30.9%	18	16.4%	**0.031**
	No	38	69.1%	92	83.6%	
Zika infection	Yes	27	46.6%	9	7.8%	**<0.001**
	No	31	53.4%	107	92.2%	
Diagnosis of zika by a health professional	Yes	18	81.8%	6	66.7%	0.360
	No	4	18.2%	3	33.3%	
Gestation month of zika diagnosis		3	2.0–4.0	5	3.0–6.0	0.246
What was the severity of zika?	Low	14	63.6%	7	87.5%	0.360
	Moderate	4	18.2%	1	12.5%	
	High	4	18.2%	0	0.0%	
Internment due to zika	Yes	0	0.0%	1	11.1%	0.120
	No	21	100.0%	8	88.9%	
Had dengue fever	Yes	1	1.8%	1	0.9%	0.606
	No	56	98.2%	115	99.1%	
Had chikungunya	Yes	1	1.8%	0	0.0%	0.153
	No	56	98.2%	116	100.0%	
Had rubella	No	57	100.0%	116	100.0%	–
Had toxoplasmosis infection	Yes	2	3.5%	3	2.6%	0.734
	No	55	96.5%	113	97.4%	
Had cytomegalovirus infection	Yes	2	3.5%	0	0.0%	**0.042**
	No	55	96.5%	116	100.0%	
Had herpes	Yes	2	3.5%	1	0,9%	0.210
	No	55	96.5%	115	99.1%	
Had syphilis	Yes	1	1.8%	1	0.9%	0.606
	No	56	98.2%	115	99.1%	
Had AIDS	No	56	100.0%	116	100.0%	–
Had hypertension	Yes	3	5.5%	18	15.8%	0.056
	No	52	94.5%	96	84.2%	
Had eclampsia	Yes	1	3.3%	2	3.3%	1.000
	No	29	96.7%	58	96.7%	
Had preeclampsia	Yes	0	0.0%	6	10.0%	0.078
	No	29	100.0%	54	90.0%	
Had diabetes	Yes	1	1.8%	6	5.3%	0.292
	No	54	98.2%	108	94.7%	
Had kidney disease	Yes	1	3.3%	5	8.3%	0.370
	No	29	96.7%	55	91.7%	
Had anemia	Yes	12	21.1%	23	20.2%	0.534
	No	45	78.9%	91	79.8%	
Had the flu	Yes	18	31.6%	44	38.3%	0.390
	No	39	68.4%	71	61.7%	
Had diarrhea	Yes	8	14.0%	7	6.1%	0.082
	No	49	86.0%	108	93.9%	
Had an allergy	Yes	5	8.9%	5	4.3%	0.225
	No	51	91.1%	111	95.7%	
Had asthma	Yes	1	3.3%	1	1.7%	0.613
	No	29	96.7%	59	98.3%	

### Exposures during pregnancy

From all the evaluated exposures, only the exposure to radiation before and during pregnancy had different odds between the groups (0.010 and 0.036 for radiography and tomography, respectively). Contact with chemical agents, repellents, and other substances did not present significant p values (Table 3).

**Table 3:** Ratios of exposures of the mother during pregnancy in cases and controls.

		Condition				p value

		Case		Control		

		n or median	% or interquartile	n or median	% or interquartile	

Worked during pregnancy	Yes, outside home	20	35.1%	31	27.2%	0.235
	Yes, at home	8	14.0%	10	8.8%	
	No	29	50.9%	73	64.0%	
Contact with ink	Yes, during pregnancy	16	27.6%	35	30.7%	0.822
	Yes, before	1	1.7%	1	0.9%	
	No	41	70.7%	78	68.4%	
Contact with varnishes	Yes, during pregnancy	8	13.8%	9	7.9%	0.168
	Yes, before	1	1.7%	0	0.0%	
	No	49	84.5%	105	92.1%	
Contact with solvents	Yes, during pregnancy	8	13.8%	12	10.4%	0.291
	Yes, before	1	1.7%	0	0.0%	
	No	49	84.5%	103	89.6%	
Contact with tails	Yes, during pregnancy	9	15.8%	14	12.2%	0.637
	Yes, before	0	0.0%	1	0.9%	
	No	48	84.2%	100	87.0%	
Contact with repellents	Yes, during pregnancy	20	34.5%	55	47.8%	0.095
	No	38	65.5%	60	52.2%	
Contact with pesticides	Yes, during pregnancy	4	7.0%	7	6.1%	0.814
	No	53	93.0%	108	93.9%	
Contact with poisons	Yes, during pregnancy	7	12.3%	19	16.8%	0.438
	No	50	87.7%	94	83.2%	
Contact with pesticides	Yes, during pregnancy	0	0.0%	2	1.8%	0.314
	No	57	100.0%	112	98.2%	
Contact with hair dye	Yes, during pregnancy	8	13.8%	21	18.4%	0.443
	No	50	86.2%	93	81.6%	
Contact with enamels	Yes, during pregnancy	33	56.9%	67	59.3%	0.953
	Yes, before	1	1.7%	2	1.8%	
	No	24	41.4%	44	38.9%	
Contact with capillary smoothing with ammonia	Yes, during pregnancy	2	3.4%	6	5.3%	0.667
	Yes, before	0	0.0%	1	0.9%	
	No	56	96.6%	107	93.9%	
Contact with capillary straighteners without ammonia	Yes, during pregnancy	2	3.4%	6	5.2%	0.672
	Yes, before	0	0.0%	1	0.9%	
	No	56	96.6%	108	93.9%	
Performed radiographs	Yes, during pregnancy	6	10.5%	2	1.8%	**0.010**
	No	51	89.5%	112	98.2%	
Performed CT scans	Yes, during pregnancy	0	0.0%	1	0.9%	**0.036**
	Yes, before	3	5.4%	0	0.0%	
	No	53	94.6%	113	99.1%	
Performed Magnetic	Yes, during pregnancy	2	4.8%	0	0.0%	0.074
Resonance	Yes, before	1	2.4%	1	1.0%	
Imaging	No	39	92.9%	98	99.0%	
Smoked during pregnancy	Yes, all the days	1	17%	5	4.3%	0.272
	Yes, some days	2	3.4%	1	0.9%	
	No, stopped smoking	3	5.2%	2	1.7%	
	No, never smoked	52	89.7%	108	93.1%	
Number of cigarettes per day		15	10.0–20.0	7	4.0–10.0	0.333
Alcohol consumption during pregnancy	Yes, a little	5	8.8%	11	9.5%	0.999
	Yes, moderate	1	1.8%	2	1.7%	
	Yes, a lot	1	1.8%	2	1.7%	
	No	50	87.7%	101	87.1%	

### Multivariate analysis

After the multivariate analysis, the variable infection by zika virus remained independently associated, with a p-value of 0.018 and odds ratio of 14.68 (1.59–134.83) (Table 4).

**Table 4:** Multivariate analysis of the determinants of microcephaly.

	B	S.E.	Adjusted OR	Adjusted OR CI 95%		p-value

				Inferior	Superior	

Zika virus	2.686	1.131	14.680	1.598	134.833	0.018
Muscle pain	0.480	0.711	1.617	0.401	6.517	0.499
Joint pain	0.731	0.602	2.078	0.638	6.768	0.225
Skin changes	–0.321	0.905	0.725	0.123	4.277	0.723
Sting during pregnancy	0.115	0.796	1.122	0.236	5.345	0.885
Radiography	0.261	0.908	1.298	0.219	7.692	0.774
CT Scan	–0.434	0.899	0.648	0.111	3.777	0.630

Block 1: Zika virus, muscle pain, joint pain, skin changes, stings during pregnancy. Block 2: Radiography, CT scan.

## Discussion

The results of this case-control study indicate that infection by zika virus diagnosed by a physician in the first trimester was, independently, the risk factor most strongly associated with microcephaly, with an adjusted OR of 14.68 (95% CI 1.59–134.83) and high statistical significance (p < 0.001) despite the small sample size. The prevalence of zika virus infection in mothers of children born with microcephaly, confirmed by diagnostic methods suggested by the WHO, was 46.6%, against only 7.8% of mothers who had healthy children during the same period, as suggested by case series [16] and ecological [15] studies and also from another case-control study, in a state near Ceará [26].

Symptoms of the infectious arboviruses syndrome, mainly occurring during the first trimester of pregnancy, associated with skin rash or joint pain, have shown an association with the development of microcephaly in the bivariate analysis. Additionally, there was a higher risk of mosquito bites during pregnancy reported by the mothers of children with microcephaly.

A preliminary laboratory case-control study conducted in another state in the northeastern region of Brazil identified a high prevalence of zika virus infection in mothers of infants with microcephaly (80%) and the mothers of controls (64%) using RT-PCR and new serological methods. However, zika virus infection did not occur in any children in the control group [27].

This study contributed to the investigation of epidemiological factors, not only the maternal infections and exposures already studied, but also identified factors such as radiation exposure. Additionally, it found an association of microcephaly with maternal exposure to radiological examinations during pregnancy, although this association did not remain after controlling for confounders. Other authors [28,29] have reported this association with radiation and congenital disabilities; however, this is not fully established. We have not yet found mention in the literature of studies that have specifically evaluated this association.

This study was validated by the difference in the means of cephalic perimeter (CP) among the case and control groups. The average CP of the children with microcephaly was below that found by Rocha et al. (2016), who performed an evaluation study to check the normality parameters of CP in children born at term in the Brazilian Northeast, before the context of the epidemic of microcephaly [30]. Other biological criteria evaluated ensured uniformity between the groups in addition to CP, including gestational age and sex.

Despite alcohol abuse and smoking during pregnancy being causes of congenital malformations [4,31], no association with microcephaly was found in this study.

As Von der Hagen et al. (2014) discussed, 41% of microcephaly cases are idiopathic [11]. The absence of a significant correlation in this study between the classic factors of teratogenesis, such as alcohol or tobacco [32], led to an investigation of other causes, such as the epidemic of the zika virus.

Microcephaly may not be the only outcome in children infected with zika virus, and other neurological disorders can develop after birth [33,34].

This study was developed with a community design that is well-suited for other risk factors beyond zika infections but with their environmental and socioeconomically correlated factors. Additionally, the emergency situation requires an exploratory study of a broad spectrum. In this sense, we evaluated more than 200 variables, including various epidemiological factors.

### Limitations

The memory bias was considered during the data collection using health records whenever possible. Confirmation bias is possible, but there was great diffusion in Brazil of the possible association between zika virus and microcephaly, which has generated a widespread awareness further than the mothers of infants with microcephaly.
